# Excretion and viability of SARS-CoV-2 in feces and its association with the clinical outcome of COVID-19

**DOI:** 10.1038/s41598-022-11439-7

**Published:** 2022-05-05

**Authors:** Cristina Cerrada-Romero, Judith Berastegui-Cabrera, Pedro Camacho-Martínez, Josune Goikoetxea-Aguirre, Patricia Pérez-Palacios, Sonia Santibáñez, María José Blanco-Vidal, Adoración Valiente, Jorge Alba, Regino Rodríguez-Álvarez, Álvaro Pascual, José Antonio Oteo, José Miguel Cisneros, Jerónimo Pachón, Inmaculada Casas-Flecha, Elisa Cordero, Francisco Pozo, Javier Sánchez-Céspedes

**Affiliations:** 1 Unit of Infectious Diseases, Microbiology, and Preventive Medicine, Virgen del Rocío University Hospital, Seville, Spain; 2 Institute of Biomedicine of Seville (IBiS), Virgen del Rocío and Virgen Macarena University Hospitals/CSIC/University of Seville, Seville, Spain; 3 Departament of Infectious Diseases, Cruces University Hospital, Bizkaia, Spain; 4 Unit of Infectious Diseases, Microbiology, and Preventive Medicine, Virgen Macarena University Hospital, Seville, Spain; 5 Department of Infectious Diseases, San Pedro University Hospital-CIBIR, Logroño, La Rioja Spain; 6 Department of Medicine, University of Seville, Sevilla, Spain; 7 Flu and Respiratory Virus Unit, National Centre of Microbiology, Institute of Health Carlos III (ISCIII), Ctra. Majadahonda a Pozuelo, Km. 2, 28220 Madrid, Spain; 8 Viral Diseases and Infections in Immunodeficiencies Research Group, Institute of Biomedicine of Seville (IBiS), Virgen del Rocío University Hospital/CSIC/University of Seville, Manuel Siurot s/n, 41013 Seville, Spain

**Keywords:** Viral infection, SARS-CoV-2

## Abstract

The main objective was to evaluate the viability of the SARS-CoV-2 viral particles excreted in stools. In addition, we aimed to identify clinical factors associated with the detection of SARS-CoV-2 RNA in feces, and to determine if its presence is associated with an unfavorable clinical outcome, defined as intensive care unit (ICU) admission and/or death. A prospective multicenter cohort study of COVID-19 adult patients, with confirmed SARS-CoV-2 infection by RT-PCR assay in nasopharyngeal (NP) swabs admitted to four hospitals in Spain, from March 2020 to February 2021. Sixty-two adult COVID-19 patients had stool samples collected at admission and/or during the follow up, with a total of 79 stool samples. SARS-CoV-2 RNA was detected in stool samples from 27 (43.5%) out of the 62 patients. Replicative virus, measured by the generation of cytopathic effect in cell culture and subsequent RT-PCR confirmation of a decrease in the Ct values, was not found in any of these stool samples. Fecal virus excretion was not associated with the presence of gastrointestinal symptoms, or with differences in the evolution of COVID-19 patients. Our results suggest that SARS-CoV-2 replicative capacity is null or very limited in stool samples, and thus, the fecal–oral transmission of SARS-CoV-2 as an alternative infection route is highly unlikely. In our study, the detection of SARS-CoV-2 RNA in feces at the beginning of the disease is not associated with any clinical factor nor with an unfavorable clinical outcome.

## Introduction

The primary routes of transmission of severe acute respiratory syndrome coronavirus 2 (SARS-CoV-2) known so far are through respiratory droplets, aerosols and close person-to-person contact^1^. It is well known that other human coronaviruses, such as SARS-CoV and MERS-CoV, are excreted in the stools of infected patients and remain viable under conditions that could facilitate fecal–oral transmission^2^, which still constitute an uncertain factor in the case of SARS-CoV-2 infection. Six studies, recently summarized^3^, including 371 adult COVID-19 patients, had reported detection of SARS-CoV-2 in stool and rectal swab samples, with frequencies ranging from 15.3% to 81.8%. Additionally, some studies have reported data of six cases suggesting the presence of viable SARS-CoV-2 in stool samples^4–7^. Recently, in a patient with rectal surgery performed three days before symptoms onset of COVID-19, direct evidence of active SARS-CoV-2 replication in intestinal tissue has been reported^8^, suggesting SARS-CoV-2 fecal–oral transmission.

In this regard, we conducted a prospective multicenter cohort study of COVID-19 adult patients aimed to evaluate if viral fecal excretion could contribute to viral transmission. In addition, our objective was to identify clinical factors associated with the detection of SARS-CoV-2 RNA in feces, and to determine if its presence is associated with an unfavorable clinical outcome, defined as intensive care unit (ICU) admission and/or death.

## Results

Sixty-two patients were included, with a median age of 59 (51–68) years, and 38 (61.2%) of them were male. Nineteen patients (30.6%) had some chronic underlying conditions, with the most frequent being solid organ transplantation (n = 14, 73.6%; 9 kidney, 4 liver, 1 heart).

### SARS-CoV-2 RNA detection in stool samples

The 62 patients had stool samples collected at admission and/or during the follow up, until four months after the onset of symptoms, with a total of 79 stool samples. Patients with stool samples collected during 3rd and 4th weeks (n = 14) or between the 2nd and the 4th months (n = 9) were solid organ transplant recipients in 7 (50.0%) and 8 (88.9%) of the cases, respectively.

**Figure 1:**
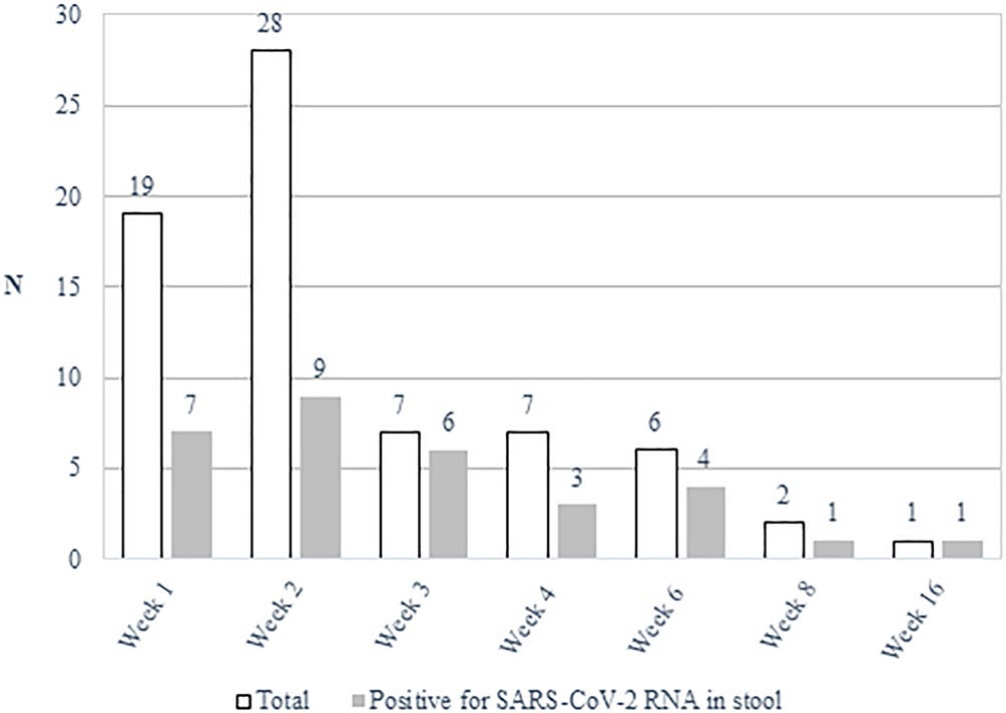
Frequency of samples (N) evaluated and with positive detection by RT-PCR of SARS-CoV-2 RNA in stools, according to the time of follow up after symptoms onset.

Twenty-seven (43.5%) out of the 62 patients had SARS-CoV-2 RNA detected in feces. Within the first and second weeks after symptoms onset, 7 (36.8%) and 9 (32.1%) out of 19 and 28 patients, respectively, had RT-PCR positive stools samples for SARS-CoV-2. The frequencies of patients with SARS-CoV-2 detection depending on the time from the symptom’s onset are shown in Fig. 1. The Ct values of the positive stool samples collected in the two first weeks ranged from 24.5 to 39.6 (median 31.2) in the first week and from 27.2 to 39.1 (median 34.5) in the second week. The Ct values in the nasopharyngeal (NP) swabs at the same periods ranged from 17 to 30 (median 21.7) and from 11.3 to 37.8 (median 33.3), respectively; the CT values were lower in NP swabs than in stool samples in the seven patients analyzed in the first week, and in six out of 9 patients in the second week of the disease. However, NP swabs were negative in 3 out of 9 and in 3 out of 6 patients analyzed in the 3–4 weeks and 2–4 months after symptoms onset.

Thirty-one stool samples with SARS-CoV-2 detected by RT-PCR (Ct values ranging from 24.3 to 39.6), corresponding to 27 patients, were analyzed for virus isolation. Cytopathic effect was observed in cultures inoculated with 24 (77.4%) of the samples after 3–7 days of incubation. Supernatants of these positive cell cultures were sub-cultured and a RT-PCR of SARS-CoV-2 performed after 7 days of incubation or when cytopathic effect (CPE) was observed. However, none of the tested supernatants was positive for SARS-CoV-2 in the RT-PCR.

### Baseline clinical characteristics and outcomes

Baseline clinical characteristics and outcomes of the 47 patients with stool samples collected in the first two weeks after symptoms onset, are detailed in Table 1. Sixteen (34%) of these patients showed a positive identification of SARS-CoV-2 in stool samples. There were no differences regarding age, sex, and chronic underlying diseases depending on the presence or absence of SARS-CoV-2 identification in stool samples. Moreover, no symptoms nor signs were more frequent in patients with SARS-CoV-2 RNA in stool samples compared to those without it, including gastrointestinal symptoms. At admission, pneumonia was more frequent in patients without SARS-CoV-2 detection in stool samples (100% *vs.* 75%, *P* = 0.01). In the analytical data, there were no differences between patients with and without SARS-CoV-2 RNA in stool samples. In addition, there was not differences in the viral load in NP swabs between patients with positive (7.10 Log10 copies/mL [IQR 4.46–9.07]) and negative identification (7.81 Log10 copies/mL [IQR 6.13–9.01]) of SARS-COV-2 RNA in stool samples collected during the first two weeks (Table 1).

**Table 1:** Demographics, chronic underlying diseases, baseline clinical characteristics, and outcomes of patients with stool samples collected in the first two weeks after symptoms onset.

Variables, *N* (%)	With RNA in feces (*N* = 16)	Without RNA in feces (*N* = 31)	*P-*value^b^
Age (median [IQR])	60 (47–71)	62 (52–70)	0.544
Male sex	8 (50.0%)	21 (67.7%)	0.236
Chronic kidney disease	1 (6.3%)	4 (12.9%)	0.648
Chronic liver disease	1 (6.3%)	1 (3.2%)	1.000
Connective tissue disease	1 (6.3%)	0 (0%)	0.340
Solid organ transplantation	3 (18.8%)	1 (3.2%)	0.108
Diarrhea	5 (31.3%)	7 (22.6%)	0.725
Vomiting	0 (0%)	0 (0%)	[..]
Cough	11 (68.8%)	19 (61.3%)	0.614
Odynophagia	2 (12.5%)	1 (3.2%)	0.264
Headache	6 (37.5%)	7 (23.3%)	0.328
Anosmia	3 (18.8%)	6 (19.4%)	1.000
Dysgeusia	4 (25.0%)	8 (25.8%)	1.000
Fever	9 (56.3%)	23 (74.2%)	0.211
SpO_2_ < 95% at diagnosis	3 (18.8%)	13 (43.3%)	0.095
qSOFA ≥ 2	0 (0%)	2 (6.5%)	0.541
Pneumonia	12 (75.0%)	31 (100%)	0.010
Remdesivir^a^	2 (12.5%)	14 (45.1%)	0.314
Leucocytes > 11,000/μL	1 (6.7%)	1 (3.2%)	1.000
Neutrophils > 7,500/μL	3 (18.8%)	3 (9.7%)	0.395
Lymphocytes < 1000/µL	9 (56.3%)	14 (45.2%)	0.471
Creatinine > 1.3 mg/dL	4 (25.0%)	4 (12.9%)	0.416
CRP > 100 mg/L	2 (12.5%)	6 (20.0%)	0.694
LDH > 300 UI/L	4 (26.7%)	13 (43.3%)	0.277
SARS-CoV-2 RNAemia	1 (6.7%)	2 (7.1%)	1.000
SARS-CoV-2 RNA in NP swabs (Log_10_ copies/mL)	7.10 (4.46–9.07)	7.81 (6.13–9.01)	0.351
ARDS	1 (6.3%)	1 (3.2%)	1.000
IMV	0 (0%)	1 (3.2%)	1.000
ICU admission and/or death	1 (6.3%)	1 (3.2%)	1.000
**Basal score**			
Moderate	16 (100%)	31 (100%)	[..]
Severe	0 (0%)	0 (0%)	[..]
**Final score**			
Moderate	15 (93.8%)	30 (96.8%)	1.000
Severe	1 (6.3%)	1 (3.2%)	1.000

*CRP* C-reactive protein, *LDH* lactate dehydrogenase, *NP* nasopharyngeal, *ARDS* Acute Respiratory Distress Syndrome, *IMV* invasive mechanical ventilation, *ICU* intensive care unit.

^a^Remdesivir administered before the collection of stool samples.

^b^Two-tailed test.

The severity of COVID-19 according to the outcome measure score proposed by the World Health Organization^8^ for patients admitted to the hospital and with stool samples collected in the first two weeks of disease, both at admission and discharge, is shown in Table 1.

## Discussion

In this study, SARS-CoV-2 RNA was identified in stool samples in almost half of adult COVID-19 patients. The stool samples collected in the two first weeks after symptoms onset were positive in one out of three patients, and a subset of patients, mostly solid organ transplantation recipients, had prolonged SARS-CoV-2 RNA excretion, until week 16th. However, we did not found replicative virus in those stool samples with SARS-CoV-2 RNA identified by RT-PCR.

Potential SARS-CoV-2 infection in the gastrointestinal tract has been discussed in regard to the expression of angiotensin-converting enzyme 2 (ACE2) and TMPRSS2, involved in SARS-CoV-2 entry mechanism in the intestine^3^. In addition, it has been reported the presence of virus particles in the intestinal tissues suggesting an active gastrointestinal viral replication^8^. The potential fecal–oral transmission of SARS-CoV-2 lies in the fact that prolonged viral shedding can occur in stool^6^. However, while prolonged viral shedding in stool has been noted, the detection of viral genetic material or complete viral particles in stool does not necessarily indicate that viable infectious virions are present in this fecal material or that the virus can or has spread through fecal transmission. To date, a small number of studies have addressed the former directly^9,10^ and it is presently believed that SARS-CoV-2 may have a low infective dose^10^, implying that low viral loads in stool could still be a concern for transmissibility.

Furthermore, in nasopharyngeal samples, it was observed an association between Ct values and sample infectivity, as defined by grow in cell culture^11^. It was noticed that infectivity is significantly reduced when RT-PCR Ct values are greater than 24^11^. In our study, positive samples with Ct values ranging from 24.3 to 39.6 were found, and SARS-CoV-2 was not successfully cultivated from any stool sample, as it was also reported by Wölfel et al.^12^. Possible explanations could be that the fecal material is not optimal for the virus survival and/or that the SARS-CoV-2 detected in feces represents only residual genetic material and not replicative viral particles.

Regarding the clinical meaning of excretion SARS-CoV-2 in stools, our analysis of the clinical variables agree with those reported by other authors^2^, suggesting that virus excretion in feces is not associated with the presence of gastrointestinal symptoms, or with differences in the evolution of COVID-19 patients. In addition, SARS-CoV-2 RNA detection in stool samples was not associated with the outcome according to the WHO Clinical Progression Scale^13^ nor with the ICU admission and/or death. Demographics, clinical characteristics, inflammatory biomarkers, and NP viral load or SARS-CoV-2 RNAemia did no differ between patients with *vs*. without SARS‐CoV‐2 RNA detection in feces.

Finally, it is worth mentioning some limitations of the results obtained in our study. First, our results and conclusion are based in a small sample size which may not be generalizable to the wider population and preclude the ability to undertake inferential statistics; thus, only a descriptive analysis was performed. Moreover, baseline data for some patients were missing and we only took into consideration those patients who had samples collected during the two first weeks after symptoms onset to analyze the impact on clinical outcomes.

In summary, our study suggests that the fecal–oral transmission of SARS-CoV-2 if any is negligible as an alternative infection route. Moreover, in our experience, the detection of SARS-CoV-2 RNA in feces at the beginning of the disease is not associated with any clinical factor nor with unfavorable clinical outcome.

## Methods

### Study design and patients

This study was conducted in adult COVID-19 patients, with confirmed SARS-CoV-2 infection by RT-PCR assay in nasopharyngeal (NP) swabs^14^ admitted to four hospitals in Spain, from March 2020 to February 2021. After the informed consent of patients, stool samples were collected in collection tubes and kept at 4 °C until their processing before four days, after admission or in the follow up after hospital discharge in cases with prolonged disease. The study was approved by the Ethics Committee of Virgen del Rocío and Virgen Macarena University Hospitals (C.I. 0842-N-20), as well as by the Euskadi (CEIm-E, C.I.: PI2020081) and the La Rioja (CEImLAR, C.I.: NO EPA-177) Ethics Committee for Research with Medicines, and complied the Declaration of Helsinki.

### RNA extraction and real time RT-PCR

All stool samples were pretreated before RT-PCR test and virus isolation. Briefly, a representative amount of 2 g of each stool sample was homogenized in 10 mL of PBS (Thermo-Fisher) containing 100 µL/mL penicillin (BioWhittaker) and 100 µL/mL streptomycin (BioWhittaker). After a vigorous vortexing for about 2 min, samples were centrifuged for 20 min at 3000×*g* in a Kubota rotor RS-720 (Kubota, Tokyo, Japan) and the resulting supernatant was filtered through a 0.45 µm membrane filter (Millipore) by a second centrifugation at 12,000×*g* for 4 min. Then, a total of 200 µL of stool sample was mixed with 200 µL of lysis buffer containing guanidine thiocyanate for external lysis. Total RNA was extracted by using the Mini Kit viral RNA (QIAGEN, Germany) with a spin column method according to the manufacturer instructions. An exogenous internal control was used to assess the efficiency of the RNA extraction process by adding 20 µL of a SARS-CoV-2 negative human nasopharyngeal exudate sample. The extracted RNA was amplified using a RT-PCR method based on the method designed by Corman et al.^15^ for the specific amplification of the E gene using the SARS-CoV-2 One-Step RT-PCR Kit (NZYTech).

### Virus isolation

SARS-CoV-2 isolation was attempted on Vero E6 cells using treated stool samples testing positive by RT-PCR, irrespective of the Ct values. A total of 50 µL from the original undiluted sample and 50 µL of two-fold serial dilutions (1:2 to 1:16) were inoculated in duplicate into 96-well microplates containing Vero E6 monolayers and were incubated at 37 °C without CO_2_. The cultures were observed daily for cytopathic effect (CPE) for 7 days. After this incubation, a subculture was performed in those samples that showed CPE, and the presence of SARS-CoV-2 was confirmed by RT-PCR in the supernatant of the cell cultures. To consider supernatants positive for SARS-CoV-2 replication a decrease in their Ct value compared to the Ct value in the original stool sample had to be found.

### SARS-CoV-2 quantification in nasopharyngeal swabs

After the extraction of SARS-CoV-2 total RNA using the EZ1 Virus Mini Kit v2.0 (Qiagen Inc., Valencia, CA, USA), genomic RNA was amplified in a LightCycler 96 Instrument (Roche, Germany) using CDC 2019-Novel Coronavirus (2019-nCoV) Real-Time RT-PCR Diagnostic Panel and the GoTaq® Probe 1-Step RT-qPCR System (Wisconsin, USA). The Quantitative Synthetic SARS-CoV-2 RNA: ORF, E, N kit (ATCC, VA, USA) was run for each NP sample and Ct values of the samples were interpolated into the curve to calculate the viral load in Log_10_ copies/mL^14^.

### Statistical analysis

A descriptive analysis of data was performed. Categorical variables were presented as frequencies and percentages and continuous variables as median and interquartile range (IQR) or ranges. We used the *χ*^2^-test, Fisher’s exact test, and Mann–Whitney U test to compare between-group differences, with a two-tailed *P* < 0.05 was considered significant.
